# Digital home-based multimodal prehabilitation of colorectal cancer patients prior to surgery (the dHOPE study): a non-inferiority clinical trial protocol

**DOI:** 10.3389/fdgth.2025.1609678

**Published:** 2025-10-08

**Authors:** Guro Kleve, Chiara Dalla Santa, Felicia Kristiansen, Marita Tegnander, Victoria Buch, Cecilie Steen Hagemann, Marianne Helgheim, Parasto Engene, Henri Pesonen, Maiken Kojen Kleveland, Greta Lyster Hverven, Tormod Skogstad Nilsen, Mathyn Adrianus Marinus Vervaart, Helle Aanesen, Kari Thune Uglane, Mona Bjelland, Raphaël Zory, Frédéric Chorin, Victoire Cardot-Ruffino, Eva Ester Molina Beltran, Cindy Neuzillet, Mette Soerensen, Juulia Jylhävä, Irantzu Anzar, Valentin André Normand, Marius Myrstad, Arne Klungland, Hilde Loge Nilsen, Rune Ougland

**Affiliations:** ^1^ Department of Surgery, Bærum Hospital Vestre Viken Hospital Trust, Gjettum, Norway; ^2^ Centre for Embryology and Healthy Development (CRESCO), Department of Clinical Medicine, Faculty of Medicine, University of Oslo, Oslo, Norway; ^3^ Applied Mathematics for Biomedical Research Institute, Ambr Institute AS, Oslo, Norway; ^4^ Centre for Embryology and Healthy Development (CRESCO), Department of Microbiology, Oslo University Hospital Rikshospitalet, Oslo, Norway; ^5^ Department of Internal Medicine, Bærum Hospital Vestre Viken Hospital Trust, Gjettum, Norway; ^6^ Department of Biostatistics, Faculty of Medicine, University of Oslo, Oslo, Norway; ^7^ Department of Laboratory Medicine, Vestre Viken Hospital Trust, Bærum, Norway; ^8^ Department of Physical Performance, The Norwegian School of Sport Sciences, Oslo, Norway; ^9^ Department of Sport Science and Physical Education, Faculty of Health and Sport Sciences, University of Agder, Kristiansand, Norway; ^10^ Department of Health Management and Health Economics, University of Oslo, Oslo, Norway; ^11^ Clinical Trials Unit, Oslo University Hospital, Oslo, Norway; ^12^ AKTIV Against Cancer, Oslo, Norway; ^13^ The Norwegian Cancer Society, Oslo, Norway; ^14^ Université Côte d'Azur, Centre Hospitalier Universitaire de Nice, Clinique Gériatrique du Cerveau et du Mouvement, Nice, France; ^15^ Molecular Oncology Team, UMR 144, Institut Curie, Paris, France; ^16^ Medical Oncology Department, Institut Curie, Saint-Cloud, France; ^17^ Department of Clinical Genetics, Odense University Hospital, Odense, Denmark; ^18^ The Danish Twin Registry and Epidemiology, Biostatistics and Biodemography, Department of Public Health, University of Southern Denmark, Odense, Denmark; ^19^ Department of Medical Epidemiology and Biostatistics, Karolinska Institutet, Stockholm, Sweden; ^20^ Faculty of Medicine and Health Technology and Gerontology Research Center (GEREC), University of Tampere, Tampere, Finland

**Keywords:** prehabilitation, digitalization, personalized medicine, decentralized healthcare, colorectal cancer, exercise training, nutrition

## Abstract

**Background:**

Cancer surgery is associated with risk of complications and loss of function. Vulnerability factors, such as advanced age, malnutrition, smoking, comorbidity, frailty, and low socioeconomic status increase the risk. Lifestyle intervention prior to surgery, known as prehabilitation, often include physical activity, nutritional support, psychological coaching, and smoking cessation, increase functional reserves and reduce postoperative complications. Most importantly, it prevents loss of functional capacity and dependence.

**Methods:**

The dHOPE study is a three-armed, open-labelled, parallel-group randomized controlled trial (RCT) with non-inferiority design to compare a digital home-based prehabilitation program with a hospital-based program or no organized prehabilitation. In addition, the dHOPE study aims to identify measurable parameters reflecting the effect of prehabilitation, thus preparing for future personalization of the prehabilitation programs.

**Discussion:**

The feasibility of multimodal prehabilitation is threatened by low compliance to hospital-based programs due to burdensome commuting, even in central and metropolitan areas. In sparsely populated countries, this challenge is even more pronounced. To ensure equal healthcare to all citizens regardless of address or economic situation, there is a need to transfer the prehabilitation program to the patients’ homes. Thus, the primary hypothesis of dHOPE is that a digital home-based program is not inferior to a hospital-based program. Moreover, given the patient diversity, prehabilitation must be personalized to meet individual profiles or needs. An exploratory subtask of dHOPE is to confirm the utility of clinical, genetic, and molecular factors in evaluating prehabilitation response ultimately to identify new biomarkers and develop medical software for individual risk stratification and development of personalized prehabilitation programs.

**Clinical Trial registration:**

https://clinicaltrials.gov/, identifier (NCT06231576).

## 1 Study rationale/background

**Figure 1:**
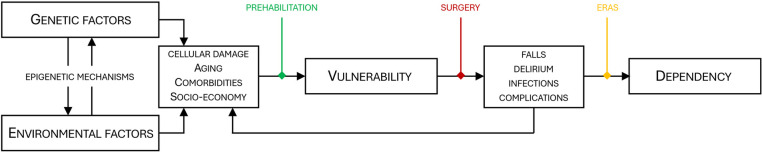
Correlation between genetic factors, environment, vulnerability and dependency. Genetics and the environment interact through epigenetic mechanisms. Over time cellular damage, aging and comorbidities are inevitable, eventually leading to increased vulnerability. Vulnerable patients have a higher risk of unfavorable outcomes after surgery, and adverse effects such as falls, delirium and complications may accelerate cellular damage and exaggerate vulnerability. For particularly vulnerable patients, the surgical trauma (red) may reduce the functional capacity below the threshold for dependency. Prehabilitation (green) and ERAS (yellow) counteract vulnerability and improve postoperative recovery thus increasing the likelihood of a self-sustained patient after surgery. ERAS, enhanced recovery after surgery.

Major cancer operations are associated with a significant risk of complications and long-term or irreversible functional loss. Vulnerability factors, such as advanced age and comorbidities, sedentary lifestyle, malnutrition, alcohol abuse, smoking, socioeconomic adversity, and lack of a next-of-kin can further increase the risk and impact the surgical outcome (1–3). Environmental influence contributes to vulnerability. In addition, yet unknown genetic and molecular factors such as genomic instability, telomere attrition, modifications of DNA (epigenetics) and RNA (epitranscriptomics), and changes to the proteome and metabolome may, individually or together, contribute to and be associated with, vulnerability (4, 5). Altered readability of the genome and transcriptome represents a sophisticated regulatory interface between genetics and environment, reflecting how environmental factors, including alterations in lifestyle, can shape gene expression and associated disease risk. Modern surgery is continuously pushing limits, and cancer surgery is offered to particularly vulnerable patients. Older individuals, even centenarians, as well as frail, multimorbid, and high-risk patients, are frequent in the operating theatre, increasing the need for personalized risk prediction and outcome assessment for surgery, cancer survival, functional levels, daily activities, and quality of life. Importantly, personalized measures to counteract pre-existing vulnerability and reduce surgical risk are required (Figure 1).

**Figure 2:**
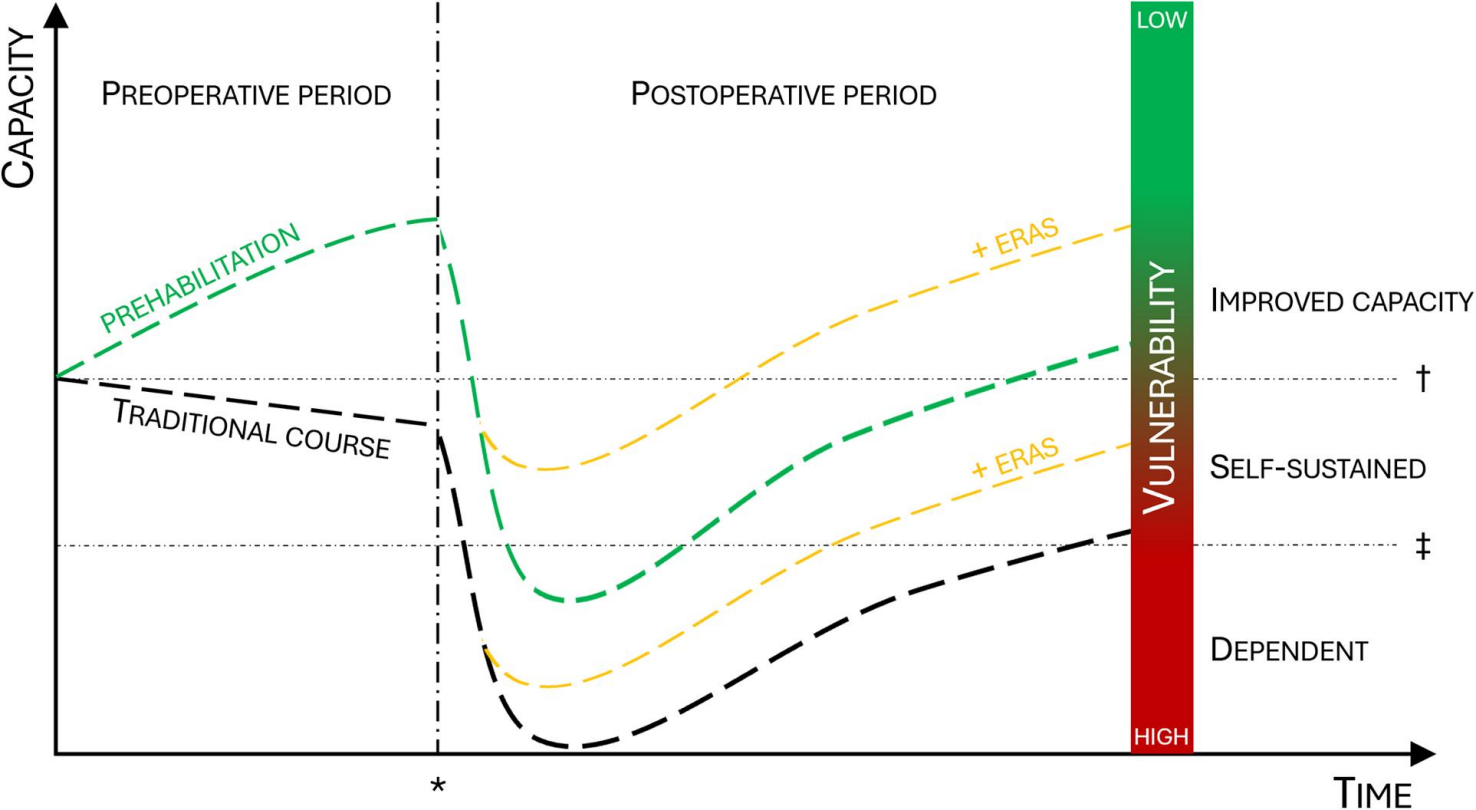
Effect of prehabilitation on surgical outcomes and postoperative level of function. When compared to the traditional course (black line) prehabilitation will increase functional capacity prior to surgery (green line) and reduce the postoperative decline. The addition of ERAS will further improve postoperative capacity (yellow line). The combination of prehabilitation and ERAS will increase the proportion of patients who stay self-sustained or improve postoperative capacity. ERAS, enhanced recovery after surgery; asterisk = time of surgery; dagger = baseline functional capacity; double dagger = threshold for dependency.

Even following the implementation of Enhanced Recovery After Surgery (ERAS) protocols for abdominal surgery (6, 7), and general optimization measures (8), cancer operations are still associated with a high rate of complications and pose a serious challenge on patients' levels of function (Figure 2). Malnutrition and sarcopenia (reduction of muscle mass and function) are major causes of morbidity and mortality in cancer patients (9). Sarcopenia has been associated with increased treatment toxicities as well as reduced progression-free- and overall survival (10). Indeed, a cohort of cancer patients exhibiting weight loss, low muscle mass and density survived 8.4 months, compared with 28.4 months in patients who had none of these characteristics (11). Aging itself, age-related diseases and the frailty syndrome increase vulnerability to stress, leading to a higher risk of disability, morbidity, and mortality (12). Frailty is commonly considered a syndrome of older individuals; however, people do not age at the same rate, and individuals with the same chronological age display great differences in biological age and divergent frailty (13–15). For reasons yet unknown, women are frailer than men despite their longer expected lifespan (16, 17). Nevertheless, advanced age and frailty are major vulnerability factors in both men and women, and the risk of a permanent reduction in functional capacity after surgery increases with pre-existing vulnerability (18, 19). Indeed, preoperative functional impairment predicts complications and mortality after cancer surgery (20, 21). Numerous studies have confirmed the association between vulnerability and slow functional recovery, and patients who do not recover swiftly after surgery may lose the opportunity to receive adjuvant anti-cancer treatment (1, 2, 18). Furthermore, when asked, the patients emphasize that functional outcomes after treatment, such as the ability to live independently, are more important than survival (22, 23). Thus, prehabilitation serves a purpose beyond survival and reduction of complications, but also by reducing dependency and increasing patient satisfaction.

Rehabilitation after surgery has been the traditional approach to counteract unfavorable outcomes after surgery. However, rehabilitation comes at a time when patients' reserves are at their lowest. Conceptually, an intervention that predates the trauma, i.e., applied before the physiological stress of surgery, is appealing and has been coined prehabilitation (24). A multimodal prehabilitation program includes intensive and coached physical exercise and optimized nutritional intake coupled with smoking cessation, physiological support and management of polypharmacy. Indeed, such programs have consistently been shown to successfully increase functional capacity (25–27), reduce complication rates in patients undergoing major abdominal surgery (28–30), lower unplanned readmissions (31), and decrease overall costs (32). Most importantly, prehabilitation can prevent the loss of functional capacity and independence (33). The observed improvement of outcomes appears to depend on a concomitant increase in protein intake to allow for lean muscle anabolism (34), and studies on physical exercise alone have shown conflicting results (35, 36). Hence, an important aspect is that complementary interventions, such as physical exercise and nutritional counseling are warranted to help sustain or rebuild muscle mass through increasing protein metabolism and decreasing catabolism (37). Cancer patients scheduled for surgery present varying levels of baseline vulnerability. While some are physically fit and exercise regularly, others are sedentary or obese. Similarly, some have no prior medical history, while others have had multiple comorbidities. It is reasonable to believe that the different patients, depending on their individual vulnerability, require tailor-made individual prehabilitation programs. However, currently, there are no biomarkers or panels of measurable indicators useful to reliably reflect individual vulnerability in predicting surgical outcomes and postoperative function. Indeed, some patients may benefit from a prolonged prehabilitation scheme, while others may be ready for surgery after a shorter prehabilitation plan. The feasibility of conventional hospital-based multimodal prehabilitation is threatened by low compliance due to burdensome commuting, even in central and metropolitan areas, as well as by the economical and logistical constraints in modern hospitals (38). To build on the known benefits of hospital-based prehabilitation programs and at the same time overcome the challenge of known constraints and low acceptance to daily long-distance commuting in a vulnerable period for surgical cancer patients, a digital approach to prehabilitation is warranted (39, 40).

This protocol describes the dHOPE study (Digital home-based multimodal prehabilitation of colorectal cancer patients prior to surgery): A three-armed, open-labelled, parallel-group randomized controlled trial (RCT) with non-inferiority design to compare digital home-based prehabilitation with a hospital-based program or no organized prehabilitation. In addition, the dHOPE study aims to identify measurable parameters reflecting the effect of prehabilitation, thus preparing for future personalization of the prehabilitation programs.

## 2 Hypotheses/objectives

The primary hypothesis is that a digital home-based prehabilitation program is not inferior to a conventional hospital-based program in improving physical fitness of patients undergoing colorectal cancer surgery. Secondly, it is hypothesized that prehabilitation reduces surgical complications and improves surgical outcomes and quality of life when compared to no prehabilitation, and that a digital prehabilitation program is cost-beneficial compared to a hospital-based program. Finally, it is hypothesized that the effect of prehabilitation imposes measurable molecular changes when compared to no prehabilitation. To test the hypotheses, the following objectives are envisioned (Table 1):

**Table 1:** List of objectives, endpoints, assessments and measure description. m5C = 5-methylcytosine; m1A = 1-methyladenine; m6A = 6-methyladenine.

Objectives	Endpoints	Assessments	Measure description
Primary			
• Physical performance	• Change in walking distance	• 6 min walking test	• Continuous score in meters
Secondary			
• Body composition • Complications/mortality • Aggregated length-of-stay • Quality of life • Healthcare resource utilization • Cost-effectiveness • Patent experience/satisfaction • Activities of daily life	• Changes to body fat/muscle distribution • Change in score • Change in days • Change in score • Change in score • Change in need/demand • Change in cost • Change in score • Change in score	• Tanita body composition monitor • Comprehensive Complication Index • Number of days • EQ-5D-5L • QLQ-C30 • Retrospective evaluation of health records • Cost per quality-adjusted life year (QALY) • GS-PEQ • Frailty index/Barthel-ADL/TUG	• Weight/fat%/muscle mass • Score (0–100) • Score (0–30) • Score (−0.59–1) • Score (0–100) • Score > 0 • Score > 0 • Score (12–60) • Score (>0/0–20/>0)
Tertiary/exploratory			
• Metabolome/proteome • Genome/epigenome • Transcriptome/epitranscriptome	• Changes in blood biomarkers • Change in DNA methylation pattern • Change in biological age and RNA methylation pattern	• Olink® Explore HT • DNA-seq and mC-seq/bisulfite-seq • RNA-seq, meRIP-seq, Induro-seq and BiTage	• Levels of 5400+ proteins in blood • Single-base resolution of m5C • Transcript levels/gene expression and distribution of m1A and m6A

### 2.1 Primary objective

The primary objective of the dHOPE study is to evaluate the effect of two different prehabilitation programs on physical fitness and compare it to usual care (no organized prehabilitation) prior to colorectal cancer surgery. The walking distance covered over a time of 6 min (6 min walking test, 6 MWT) is used as the outcome measure, and changes in walking distance is the primary endpoint to compare changes in performance capacity.

### 2.2 Secondary objectives

The three groups will be compared with respect to:

– Complications and mortality, measured by the Comprehensive Complication Index (CCI).
– Resource utilization and cost/benefit, measured by use of hospital resources, aggregated length-of-stay and need for additional healthcare support upon discharge from the hospital (such as municipal nursing homes or home visits by healthcare personnel), quality of life, measured by the EORTC Core Quality of Life questionnaire (EORTC QLQ-C30) and EuroQol Group Questionnaire 5D (EQ-5D-5L).
– Patient experience and satisfaction, measured by the Norwegian version of the Generic Short Patient Experiences Questionnaire (GS-PEQ) (41).
– Frailty and daily level of function, measured by the Norwegian versions of the Frailty index (FI) (42), Barthel Index for Activities of Daily Living (Barthel-ADL) (43) and the Timed Up and Go test (TUG) (44).
– Body weight and composition, measured by bioelectrical impedance with a Tanita body composition monitor

### 2.3 Tertiary/exploratory objectives

The three groups will be compared with respect to molecular response to prehabilitation and surgery, assessed by multi-omics changes in the blood of:

– Epigenetics (DNA)
– Transcriptomics/epitranscriptomics (RNA)
– Metabolomics/proteomics (metabolites/proteins)

Advanced machine learning techniques, including explainable artificial intelligence (XAI) methodologies will be used to integrate analyses of differences in molecular responses in relation to changes in clinical parameters and surgical outcomes.

## 3 Trial design

**Figure 3:**
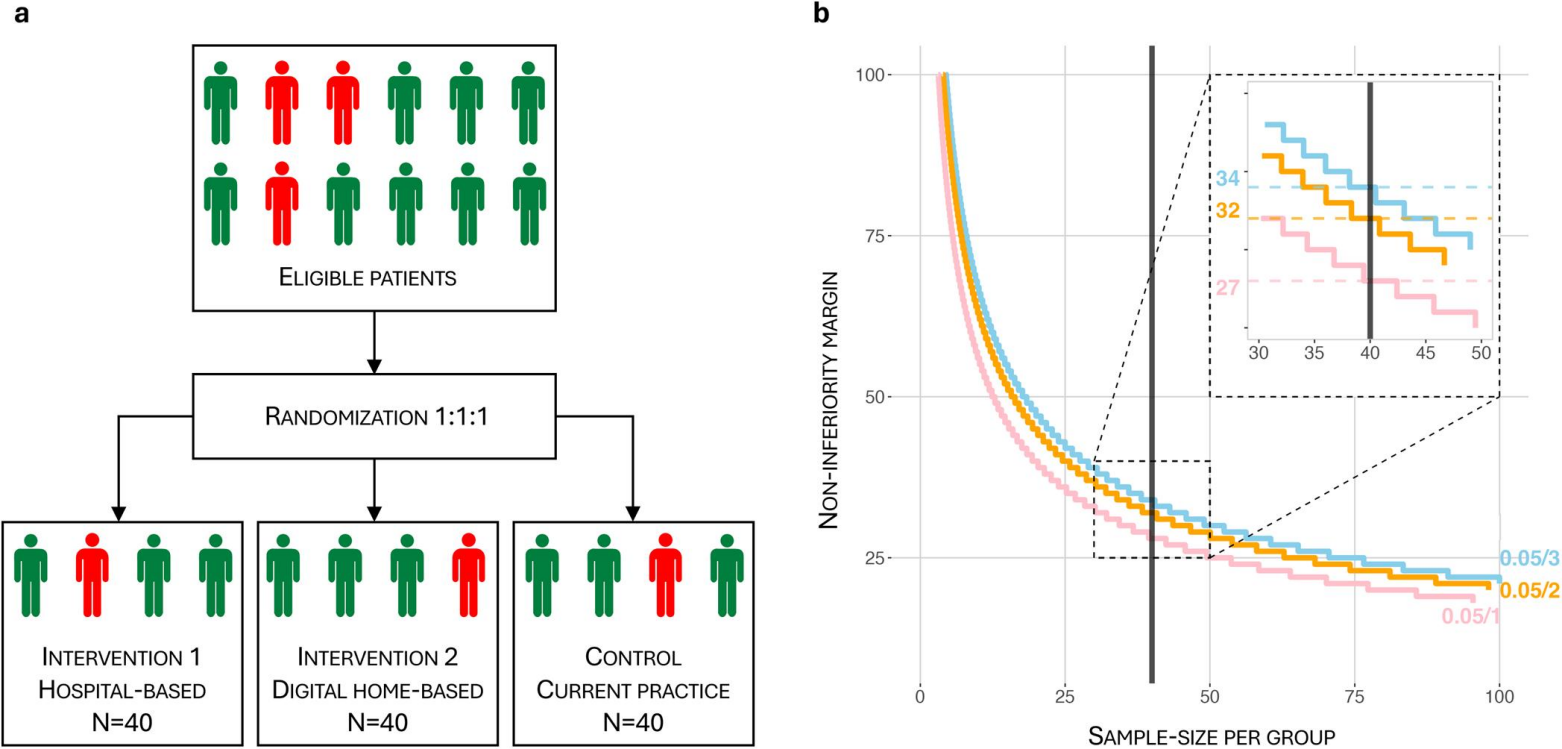
**(a)** randomization flowchart. Randomization is 1:1:1, stratified based on frailty measured by the Frailty Index (FI). FI ≥ 0.20 is considered frail, (red person) while FI < 0.20 is non-frail (green person). Each group will contain at least 25% (=10) frail patients. **(b)** Per-group sample size **(N)** vs. non-inferiority margin **(d)** for 1, 2 and 3 comparisons with corresponding Bonferroni corrections (pink, orange and blue lines respectively). The most conservative Bonferroni-corrected *α* = 0.05/3, β = 0.2 in a non-inferiority hypothesis setting gives a sample size *N* = 40 and corresponds to d = 34.

The dHOPE study is a three-armed, open-labelled, parallel-group RCT with a non-inferiority design to compare two intervention groups: A digital-home based prehabilitation group and a hospital-based prehabilitation group. The two intervention groups will be tested for superiority when compared to the control group (no organized prehabilitation). Randomization is stratified based on frailty [measured by the Frailty Index (FI)] 1:1:1 to ensure equal distribution of frailty across the groups (Figure 3a). The study includes a translational perspective by combining a clinical RCT with the search for multi-maker models, including novel biomarkers, reflecting the individual molecular response to prehabilitation and surgery.

### 3.1 Study population and setting

**Figure 4:**
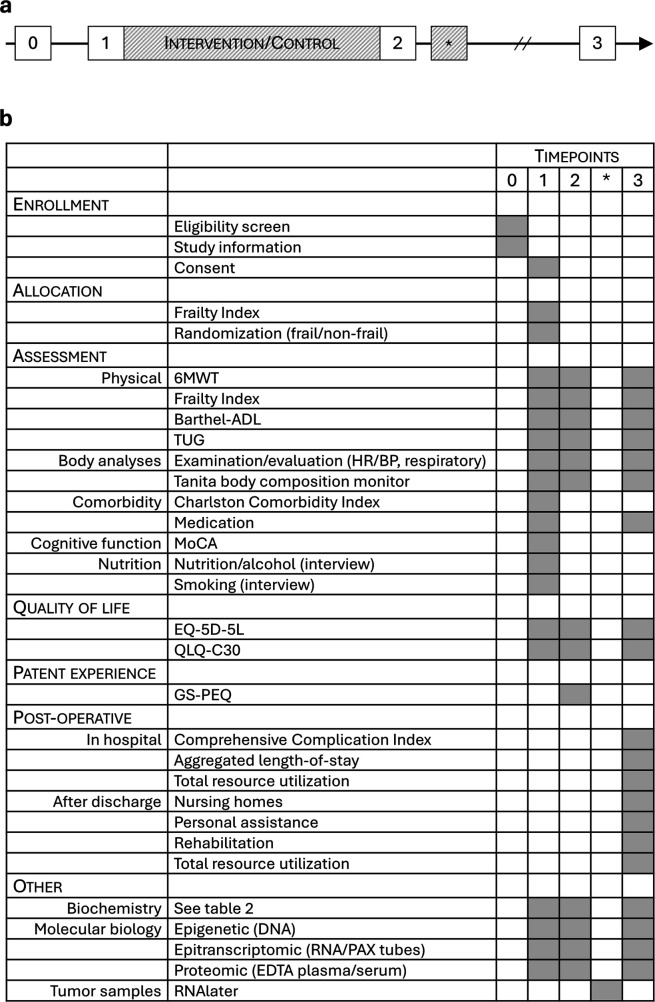
**(a)** trial timeline. 0 = MDT meeting/eligibility pre-screening; 1 = prior to intervention/control; 2 = post-intervention/control, prior to surgery; * = surgery; 3 = 4–6 weeks postoperatively/close-out. **(b)** Task table with timepoints referring to **(a)**.

Participants will be screened and recruited from the pool of patients with diagnosed colorectal cancer at Vestre Viken Health Trust (Bærum and Drammen hospitals). Upon intervention, all enrolled participants get a consultation with a surgeon, a geriatrician, and a clinical nutritionist. A complete physical examination, including cardiovascular, respiratory, gastrointestinal, and neurological system assessment will be done, as well as registration of comorbidities according to the Charlson Comorbidity Index (45). A standard panel of preoperative blood samples is analyzed by the local laboratory (Table 2). The patients are tested prior to inclusion, after intervention and 4–6 weeks after surgery (Figure 4). Blood samples for the molecular analyses are drawn by laboratory personnel at the same timepoints. Testing and randomization will be done at Bærum hospital premises by trained study nurses. Intervention is provided by physiotherapists with special education in physical activity and cancer care.

**Table 2:** List of tests, health record information and samples.

Laboratory tests	Clinical tests	Health record information	Samples for biobank
Hematology	Clinical examination	Before surgery	Genetics/epigenetics/proteomics
• Hemoglobin • Platelet count • White blood cell count	• Blood pressure • Heart rate • Oxygen saturation (SpO2) • Body weight/Height • Body mass index	• Comorbidity • Medication • Smoking • Drinking habits • Nutrition	• 3 × serum tubes (6 × 0.5 ml alliquots) • 2 × EDTA tubes (5 × 0.5 ml alliquots)
Clinical chemistry	Body composition analysis	After surgery	RNA
• C-reactive protein • Transferrin • Transferring saturation • Ferritin • Alanine transaminase • Gamma-glutamyl transpeptidase • Alkaline phosphatase • Amylase • Bilirubin • Glucose • Albumin	• Fat% • Fat mass • Muscle mass • Metabolic age	• Aggregated length-of-stay • Complications • Accumulated hospital resources • Accumulated municipal health care	• 1 × 6 ml PAX tube
Electrolytes/kidney function	Patient reported outcome measures		Specimens
• Iron • Sodium • Potassium • Calcium • Phosphate • Zinc • Magnesium • Creatinine • eGFR (creatinine) • Cystatin-C • eGFR (Cystatine-C)	• EQ-5D-5L • QLQ-C30 • GS-PEQ • Bathel-ADL		• 1 × 4 mm tumor sample (in 1 ml RNAlater)
Vitamins	Physical/mental tests		
Vitamins • Total vitamin D • Ergocalciferol (D2) • Cholecalciferol (D3)	• 6-minutes walking test • MoCA • Timed Up and Go test • Grip strength • Frailty Index		

### 3.2 Eligibility

All patients referred to Vestre Viken Health Trust (Bærum and Drammen hospitals) for colorectal cancer workup will be screened for eligibility.

#### 3.2.1 Inclusion criteria

– Planned for major gastrointestinal cancer surgery
– Fluent in Norwegian and able to understand Norwegian questionnaires
– Able and willing to consent

#### 3.2.2 Exclusion criteria

– Metastatic disease
– Previous major gastrointestinal surgery
– Physical impairment (unable to walk for six minutes or to rise independently from a chair)
– Cognitive impairment (unable to comprehend exercise programs or to comply with written and oral instructions)
– Severe comorbidity, such as cardio-pulmonary conditions that precludes exercise
– Living in remote areas hence making it impossible to participate in a hospital-based intervention group
– Being without a permanent address
– Admittance to a hospital facility for >50% of the time from diagnosis to surgery

### 3.3 Screen failure

When a potential participant has consented to participate in the study and has been screened for enrollment yet does not meet the inclusion criteria or is unwilling to participate in the scheduled intervention, it is a screen failure. Screen failures are registered.

### 3.4 Ethics and consent

Potential participants will be identified at the multidisciplinary team conference where all cancer patients are discussed. Potential participants will thereafter be contacted by phone by a study nurse. The study nurse will perform a pre-screening according to the inclusion/exclusion criteria and inform about the study aims and implications of participation in the study, including risks and benefits, thus the potential study participant will be given at least 24 h to consider enrollment. It is underscored that participation is voluntary. At the first physical meeting at the outpatient clinic, all information will be repeated by a surgeon and the written information is reviewed together with the potential participant prior to signing of the consent. There will be time for questions and discussion of any concern regarding the study. When consent is signed, their participation is stated in their electronic medical record and the participant undergoes testing and randomization. The enrolled patient will be given a copy of the consent, signed by the person authorized to obtain the informed consent. The time of surgery will be scheduled depending on the randomization; hence the intervention groups are scheduled for surgery after the prehabilitation program, while the control group patients will be scheduled for surgery within 14 days, according to the ethical approval.

The study will be conducted in accordance with the protocol and with the following:

– Consensus ethical principles derived from international guidelines including the Declaration of Helsinki and Council for International Organizations of Medical Sciences (CIOMS) international ethical guidelines
– Applicable ICH Good Clinical Practice (GCP) guidelines
– Applicable laws and regulations, including general data protection regulation (GDPR), and comply with the approvals given by the Regional Ethical Committee (Ref. No. 489036) and the local Data Protection Officer (Ref. No. 23/08551-3).

Throughout the project period, there will be regular meetings to evaluate potential ethical pitfalls. Recognizing the cultural diversity in healthcare practices and beliefs, the project will adopt a culturally sensitive approach. This involves tailoring communication strategies, considering cultural norms in participant recruitment, and acknowledging the potential impact of cultural factors on treatment decisions and outcomes. By doing so, the research seeks to enhance the applicability and acceptance of biomarker-based decision-making across diverse cultural contexts. While the primary focus is on cancer treatment outcomes, the dHOPE study will also consider the environmental impact through devising an environment-friendly digital home-based prehabilitation that minimizes emissions compared to the traditional hospital-based programs that require commuting.

## 4 Interventions

The two intervention groups and the control group will receive:

– Standard information about planned surgery, risks, and precautions
– Counsel for smoking cessation (if applicable)
– Comorbidity and polypharmacy optimization (if applicable)
– Rehabilitation postoperatively

Information about the operation will be given by a gastrointestinal surgeon, optimization of comorbid conditions and polypharmacy (if applicable) will be done by a geriatrician. Dedicated study nurses with special knowledge of cancer care will provide counseling regarding diet and smoking cessation. Two hospital-employed physiotherapists with special education in exercise oncology, will provide the exercise intervention. Postoperative rehabilitation is done by community-based physiotherapists or personal trainers.

### 4.1 Hospital-based intervention

The hospital-based group will get the following intervention at the hospital's premises:

– Exercise: Preoperatively, supervised exercise training for 1 h per day will be prescribed, 5 days per week, for three consecutive weeks aimed to increase muscle strength and aerobic capacity. Every week will include two endurance sessions and three strength training sessions. Exercise intensity will be based on the rate of perceived exertion using the Borg scale. The Borg scale is a 15-grade scale ranging from 6 to 20, where 6 is rest/very light to 20 which is maximal intensity/very hard. During weekends, participants will be prescribed unsupervised exercise sessions. The physiotherapist will make individual adjustments to the program as required, and create a training diary together with each patient, documenting each session.
– Nutritional counseling: After a comprehensive assessment of the participant's diet, personalized nutritional advice will be provided. The main goals are to avoid perioperative malnutrition and to ensure optimal protein intake to support exercise-induced anabolism, if required by nutritional support drinks. According to the surgical nutrition guidelines, a daily protein intake of 1.2–2.0 g/kg will meet the patients’ needs (46–49).
– Psychological coaching and support: Performed as one interview during inclusion, followed by weekly phone calls by a study nurse coordinator during the intervention, focusing on exercise, nutritional intake, motivation and cessation of smoking and alcohol.

### 4.2 Digital home-based intervention

The digital home-based group will get nutritional counselling at the hospital's premises at inclusion (described above under “hospital-based intervention”). The exercise intervention will be done at the patients' homes. At inclusion the digital home-based group will be equipped with a training kit containing dumbbells and rubber bands to use at home, as well as a tablet computer. This group gets daily online appointments with a physiotherapist, and their intervention will be as follows:

– Exercise: Preoperatively, supervised exercise training for 1 h per day will be prescribed, 5 days per week, for three consecutive weeks aimed to increase muscle strength and aerobic capacity. Every week will include two endurance sessions and three strength training sessions. Exercise intensity will be based on the rate of perceived exertion using the Borg scale. The Borg scale is a 15-grade scale ranging from 6 to 20, where 6 is rest/very light to 20 which is maximal intensity/very hard. Endurance training for the digital home-based intervention group can be carried out outdoors, such as through running or walking in the areas surrounding the participants’ homes. During weekends, participants will be prescribed unsupervised exercise sessions. The physiotherapist will make individual adjustments to the program as required, and create a training diary together with each patient, documenting each session.
– Psychological coaching and support: Performed as one interview during inclusion, followed by weekly phone calls by a study nurse coordinator during the intervention, focusing on exercise, nutritional intake, motivation and cessation of smoking and alcohol.

### 4.3 Control

Participants randomized to the control group will get neither supervised exercise nor nutritional counseling, except for the scheduled testing required by the dHOPE study.

### 4.4 Outcomes

Participant timeline and time points for outcome assessments are referred to by the annotations described in Figure 4 and Table 1.

#### 4.4.1 Primary outcome

The primary outcome of the dHOPE study is to evaluate the effect of two different prehabilitation programs on physical fitness and compare it to usual care (no organized prehabilitation). The walking distance, in meters, covered over a time of 6 min (6 min walking test, 6MWT) is used as the primary outcome measure to compare changes in performance capacity (50, 51). The longer the distance walked in 6 min, the better physical performance. A change in walking distance of 34 meters is considered a clinically meaningful difference, and 34 meters was used in the power calculation of sample- and group sizes. Furthermore, 34 meters is generally accepted as a clinically important difference for change in 6 MWT of adults with pathology (52). Measure description: Continuous score in meters. Time points: Baseline (1), after intervention (2), and 4–6 weeks after surgery (3).

#### 4.4.2 Secondary outcomes

The three groups will be compared with respect to:

– Complications and mortality.
This will be measured by the Comprehensive Complication Index (CCI). CCI is based on the Clavien-Dindo classification of complications (53), and was developed to reflect the gravity of the overall complication burden on the patients. To transform Clavien-Dindo registrations into a CCI number, an online calculator will be used (https://www.cci-calculator.com/cci-calculator). This calculator provides a CCI score ranging from 0 (no burden of complications) to 100 (death from complications). A difference in CCI score of 10 is considered a clinically meaningful difference (54). Complications will be registered the first 30 days after surgery and will be recorded through a journal review/search after patient discharge. Measure description: Continuous score ranging from 0 to 100. Time points: First 30 days. Registered 4–6 weeks after surgery (3).
– Aggregated length-of-stay (a-LoS).
This is defined as the sum of postoperative nights in the hospital during index stay and any transfer-or readmission stays within 30 days. Measure description: Continuous score ranging from 1 to 30. Time points: First 30 days. Registered 4–6 weeks after surgery (3).
– Quality of Life (QoL), will be measured by the following patient-reported outcome measures:
The Norwegian version of the EORTC Core Quality of Life questionnaire (EORTC QLQ-C30). This form is designed to measure cancer patients’ physical, psychological, and social functions. The questionnaire ranges from 0 to 100; a higher score represents a higher (“better”) level of functioning or a higher (“worse”) level of symptoms. Traditionally, a difference in score of 10 is considered a clinically meaningful difference (55, 56); however later studies have revealed that results vary between disease setting and cancer type (57). In dHOPE we use 10 as the meaningful difference based on findings from QoL studies of colorectal cancer patients (58), although this is a conservative estimation (59). Measure description: Continuous score ranging from 0 to 100. Time points: Baseline (1), after intervention (2), and 4–6 weeks after surgery (3).
The Norwegian version of the EuroQol Group Questionnaire 5D (EQ-5D-5L). The EQ-5D-5L is a standardized measure of health-related quality of life. It is the most widely used health-related quality of life questionnaire in health economic evaluations. The form has 5 dimensions, each dimension represented by a value 1–5, resulting in a 1-digit number that expresses the level selected for that dimension. The digits for the five dimensions can be combined into a 5-digit number that describes the patient's health state. The health state can be transformed to an index value based on a population norm (60, 61). The index scores range from −0.59 to 1, where 1 is the best possible health state, and negative values are considered worse than death. The form also contains a visual-analog scale where respondents report their self-rated valuation of their health state on a scale of 0–100. Based on data from Australia and USA, the clinically meaningful difference for the index is 0.03 and 5 for the VAS scale (59). Measure description: Dimension number, 5 digits. Continuous index score from −0.59 to 1. Continuous VAS score from 0 to 100. Time points: Baseline (1), after intervention (2), and 4–6 weeks after surgery (3).
– Healthcare resource utilization.
Healthcare resource utilization will be measured and calculated retrospectively from the participants’ electronic health records. The accumulated use of hospital resources and information on healthcare provided by the municipalities after hospital discharge will be collected and registered. This includes, but is not limited to, the use of nurses and other healthcare providers at the patients’ homes and the use of nursing home facilities, physiotherapy, or personal assistants. Measure description: Continuous number > 0. Time points: Registered 4–6 weeks after surgery (3).
– Cost-effectiveness
A health economic evaluation will be conducted to compare the cost-effectiveness of the digital and physical prehabilitation programs with standard care from a Norwegian healthcare perspective. A within-trial analysis will leverage randomized data collected directly from the clinical trial. The primary outcome of the economic evaluation is the incremental cost-effectiveness ratio (ICER), defined as the incremental cost per quality-adjusted life year (QALY) for each intervention compared to its relevant comparator. A full incremental approach will be used to rank and compare the 3 alternative interventions. The QALY is a generic measure of health effect that captures both the length and quality of life, allowing for comparisons across different interventions. QALYs are calculated by multiplying each time interval by its corresponding utility weight, reflecting the quality of life during that interval (62). These utility weights are derived from the EQ-5D-5L health state descriptions using the Norwegian EQ-5D-5L value set (61). An Area-under-the-Curve (AUC) method will be used to calculate patient-specific QALYs over the trial follow-up period (63). The calculation of costs (euros) involves multiplying the type and frequency of resource use by their respective unit costs. These unit costs will be obtained from publicly available sources, such as reimbursement rates for outpatient services from The Norwegian Health Economics Administration (Helfo), diagnosis-related groups (DRGs) from The Norwegian Directorate of Health, and the unit cost database from the Norwegian Medical Products Agency. Measure description: ICER = ratio; cost and QALYs = continuous number > 0. Time points: Registered 4–6 weeks after surgery (3).
– Patient experience and satisfaction.
This will be measured by the Norwegian Generic Short Patient Experiences Questionnaire (GS-PEQ). GS-PEQ is a questionnaire for collecting data about user experiences across different types of services. Measure description: The questionnaire has 12 questions ranging from 1 to 5; a higher score represents a higher (“better”) experience. Time points: Registered 4–6 weeks after surgery (3).
– Frailty Index (FI).
The Norwegian translation of the Frailty Index (FI) will be used as a screening tool at inclusion, prior to randomization, to ensure equal levels of frailty across the intervention groups (42). FI will be used to measure the health status of participants as it serves as a measure of vulnerability. It evaluates several age-related health variables and calculates the proportion of deficits across these variables, resulting in an index. The index is a continuous number > 0. Generally, a change in FI of 0.03 is considered a clinically meaningful difference (64, 65). Measure description: FI < 0.1 is considered non-frail, 0.10–0.19 is pre-frail, 0.20–0.29 mild degree of frailty, 0.30–0.39 moderate degree of frailty, ≥0.4 severe frailty. Time points: Baseline (1), after intervention (2), and 4–6 weeks after surgery (3).
– Barthel Index for Activities of Daily Living (Barthel-ADL).
This form consists of 20 questions designed to measure performance across 10 dimensions of daily activities. Each performance item is rated on this scale with a given number of points assigned to each dimension. A higher number is associated with a greater likelihood of being able to live at home with a degree of independence following discharge from a hospital. The clinically meaningful difference shows some variations between studies, but in dHOPE a difference of 2 will be considered meaningful, based on data from stroke patients (66). Measure description: Numbers 0–20, 0–9 indicate dependency, 10–19 indicate moderately dependent, 20 is independent. Time points: Baseline (1), after intervention (2), and 4–6 weeks after surgery (3).
– Timed Up and Go test (TUG).
This test is widely used to evaluate balance in older adult (44, 67) and is used according to the Scandinavian guidelines in dHOPE (68). The test requires the patient to stand up from a chair, walk a short distance, turn around, return, and sit down again. The clinically meaningful difference varies across patient populations and studies (69, 70). In dHOPE the patients do the test twice, and the mean of the two registrations, in seconds, is used for calculations. We consider 2 s clinically meaningful, which is a quite stringent value (71). Generally, >20 s means no need for help, while > 30 s means that the patient requires help when moving. Measure description: Continuous score in seconds. Time points: Baseline (1), after intervention (2), and 4–6 weeks after surgery (3).
– Body weight and composition.
This is measured by bioelectrical impedance with a MC-780 Tanita body composition monitor (https://tanita.eu/mc-780ma-p). Measure description: For example, but is not limited to, body weight, body fat percentage, muscle mass, body mass index, and metabolic age. Time points: Baseline (1), after intervention (2), and 4–6 weeks after surgery (3).

#### 4.4.3 Tertiary/exploratory outcomes

To identify biomarkers reflecting a response to prehabilitation and surgery, the groups will be compared along multiple trajectories: (1) groupwise, prehabilitation vs. control, (2) individually, over time, (3) frail vs. non-frail, (4) female vs. male. The following multi-omics changes will be assessed:

– Epigenome (DNA): Whole genome profiling of methylated cytosines using the Illumina NovaSeq X Plus system with an output of up to 8 Tb on the single flow cell. Bisulfite conversion of DNA is done to detect unmethylated cytosines. Bisulfite conversion changes unmethylated cytosines to uracil during library preparation. Converted bases are identified (after PCR) as thymine in the sequencing data, and read counts are used to determine the % methylated cytosines.
– Transcriptome/epitranscriptome (RNA): Prehabilitation and surgical stress transiently affect the biological age as measured with BiTage. BiTage is an aging clock that discriminates between biological and chronological age. The estimation of biological age is required for identifying gerontogenes and assessing environmental, nutritional, or therapeutic impacts on the aging process (72). BiTage predicts biological age with an accuracy that is close to the theoretical limit and is based on sequencing results of RNA using the using the Illumina NovaSeq X Plus system. As an exploratory addition, analyses of epitranscriptomic modifications will be included. Recently, the RNA modification status of small non-coding RNA and their role in gene regulation was portrayed (73), and the role of m6A in aging and frailty has been described (74, 75). In dHOPE the same workflow will be used to explore how prehabilitation and surgery alters the RNA modification pattern.
– Metabolome/proteome: Proteomics: Olink® Explore HT (https://olink.com/products/olink-explore-ht) is designed for exploratory biomarker studies. The panel is the most comprehensive among the different panels for protein profiling and enables for quantification of 5,400+ proteins. This makes Olink® Explore HT ideal for dHOPE, namely the identification of sensitive and specific biomarkers for response to prehabilitation and surgical outcomes.

### 4.5 Sample size and power calculation

Sample sizes per group are calculated in a non-inferiority trial design setting. Sample sizes are plotted against non-inferiority margins given the standard deviation from a pilot study (sd = 43). The standard deviation calculated from the pilot dataset is used even though similar studies from Montreal report greater variability (24, 76). It is justifiable as the local pilot data set's patients most likely represent the study population better than a dataset from another country.

Curves are plotted for α = 0.05 and β = 0.2 levels, with Bonferroni correction for 1, 2 and 3 comparisons (Figure 3b). The incidence of eligible patients referred to the hospital makes it feasible to include 40 patients in each group. The dHOPE study has an estimated combined screen failure (pt 3.3) and dropout rate (pt 5.5) of approximately 15%. This is considered in the ethical approval of the trial. Thus, we screen 140 patients to ensure 40 patients in each of the three study groups. The above-mentioned parameters and the most conservative Bonferroni correction for 3 comparisons, gives a non-inferiority margin (d) of 34 meters.

### 4.6 Recruitment

See pt 3.4 for a detailed description of the recruitment process. The design of dHOPE is based on a small-scale feasibility study from 2023. This study revealed that close to 100% of eligible patients consented to participation in a prehabilitation program (<5% declined). Those who did not consent, or dropped out of the program, did so because of severe comorbidity or disease progression leading to an urgent need for surgery. The feasibility study showed that the program was resource demanding, yet feasible, and satisfaction among patients and surgeons was high. Prior to the start of dHOPE we calculated that our hospital will be able to recruit approximately 2 patients/week, hence the enrollment should be completed in about 1.5 years.

### 4.7 Assignment of intervention/randomization

The dHOPE study is a three-armed, open-labelled, parallel-group RCT, and the specific intervention to be provided to the participant will be assigned using randomization envelopes. Randomization is stratified based on frailty [measured by the Frailty Index (FI)] 1:1:1 to ensure equal distribution of frailty across the groups (Figure 3a). The FI will be calculated with 2 decimal places, and FI ≥ 0.20 is considered frail, while FI < 0.20 is non-frail. Each group will contain at least 25% (=10) frail patients. The investigator will receive blinded randomization envelopes from an external statistician, one set of envelopes for the “frail” group of patients, and one set of envelopes for the “non-frail” group of patients. The envelopes contain one of 3 outcomes i.e., control, physical prehabilitation or digital prehabilitation. The envelopes will be opened in ascending numerical order immediately prior to the start of study intervention administration for each participant, and the participant keeps their unique randomization number throughout the study. The investigator will record the date and time the envelope was opened.

## 5 Data collection and management

### 5.1 Data management

All raw data generated from the dHOPE study will be stored pseudonymized at secure servers at Oslo University Hospital and Vestre Viken Health Trust. The servers are approved for sensitive patient information. Personal data collected will be handled according to national regulations and the European data protection rules described in the General Data Protection Regulation (GDPR). Test results will be collected pseudonymized by paper and later transferred to Ledidi (https://ledidi.com/). Ledidi is a secure web-based digital platform approved by the Norwegian health authorities. The platform provides user-friendly database functionality with industry standards for logging, backups, data restoration and prevention of attacks. Moreover, it includes a statistical analyses package with data visualization tools and enables easy downloading of data to other statistical packages. The interface opens for secure interoperability with collaborators and external sources. Subsequent analyses will use software that incorporates biomarker measurements, medical records, and lifestyle questionnaires to infer patient-specific risk predictions. These predictions are generated by an eXtreme Gradient Boosting (XGBoost) model, a powerful tree-based gradient boosting algorithm. A key advantage of XGBoost is its ability to automatically generate feature importance estimates indicating the significance of each feature (e.g., specific diet, physical activity, blood biomarkers, methylation pattern) in the model's decision-making process for predicting a given outcome. Feature importance is explicitly calculated for each feature, enabling ranking and comparison, where features frequently used in key decisions have higher relative importance. Additionally, Shapley Values are utilized to provide further insights into the model's decisions. The integration of such explainable AI methods in our models aims to provide transparency and interpretability, which is crucial for sensitive health applications (77, 78). By making model decisions more understandable, these methods enable the identification of key predictive variables, such as lifestyle traits or genetic factors, across multiple patients. The identification of the most important variables contributing to the outcome prediction helps uncover potential targets for prevention in prehabilitation plans, ultimately improving patient care. Feature importance scores and Shapley values can be aggregated to provide surgeons with data-driven insights and a quantitative approach to risk stratification, enabling more accurate estimation of the risk of postoperative comorbidities.

### 5.2 Confidentiality

All study-related information will be stored securely at the study site, at secure servers or in locked file cabinets in areas with limited access. All local databases will be secured with password-protected access systems. Collected material such as, but not limited to, laboratory specimens, written reports, test results and administrative forms will be pseudonymized. The encryption key will be stored separately on a secure server accessible only for the lead investigator. Any records that contain names or other personal identifiers such as, but not limited to, the signed consent forms, will be stored separately from study records. Forms, lists, logbooks, appointment books, and any other listings that link participant ID numbers to other identifying information will be stored in a separate, locked file in an area with limited access.

### 5.3 Data availability

When legally permissible, all project results will be published as open access, with raw data, associated metadata, and a detailed description of the methodology uploaded to public repositories, according to active open science principles. The main objective is to prepare the data for sharing and integration with other personalized medicine studies across Europe. Reproducible computational pipelines in open, web-based platform for data-intensive computational research for data analyses will be used. Where data protection approvals demand it, e.g., for human sequencing data, aggregated and processed raw data can be shared. However, parts of the final dataset may be restricted due to European and national regulations regarding patient privacy. These parts may be available upon request to the lead investigator. Any request will be presented for the Data Protection Officer and the Ethical Committee for legal- and ethical evaluation. After completion of the study, all raw data will be stored for a minimum of 5 years according to §38 of the Norwegian Act on medical and health research.

### 5.4 Plan for assessment and collection of outcomes (ref pt 4.4)

#### 5.4.1 Primary outcome

The 6 MWT is conducted according to the guideline (79). The test is organized by the same study nurses in the same environment, with the same conditions every time. Distance and speed (meters/second) are registered in a logbook and later transferred to Ledidi (pt 5.1) by the lead investigator.

#### 5.4.2 Secondary outcomes

Paper versions of the questionnaires EQ-5D-5L, EORTC QLQ-C30, and GS-PEQ are filled in by the enrolled participants. The tests FI, TUG, and Barthel-ADL are done, supervised by the study nurses, on the test days (Figure 4) and the results are registered in a logbook. The study nurses are responsible for ensuring that the questionnaires are filled in properly and that tests are conducted in accordance with the protocol. Body weight and composition measurements, taken using bioelectrical impedance with a Tanita body composition monitor, are performed at the same time points, and a report is generated directly from the monitor. All written registrations are later transferred to Ledidi by the lead investigator. Complications and mortality, measured by the Comprehensive Complication Index (CCI), as well as resource utilization and cost/benefit analyses, are done retrospectively after patient discharge, at the final endpoint measurement.

#### 5.4.3 Tertiary/exploratory outcomes

– Epigenome (DNA): DNA purification, bisulfite conversion of DNA, and library preparations are done in a molecular biology laboratory at Oslo University Hospital by technicians and trained laboratory personnel. Sequencing will be done by The Norwegian Sequencing Centre (NCS). NCS is a national technology core facility with long experience in sequencing services (https://www.sequencing.uio.no/). Mapping, data processing and epigenome profiling will be performed at the Department of Microbiology at Oslo University Hospital.
– Transcriptome/epitranscriptome (RNA): Library preparation for transcriptome profiling (RNA sequencing) and methylated RNA immunoprecipitation sequencing (MeRIP-seq) to detect m1A and m6A will be done at the Department of Microbiology at Oslo University Hospital by technicians and trained laboratory personnel. Sequencing will be done by NCS. Bioinformatics and calculations after sequencing will be done by university-employed scientists with special knowledge of multi-omics analyses.
– Metabolome/proteome: Proteomics analyses will be done by the Proteomic Core Facility at Oslo University Hospital (https://www.ous-research.no/proteomics/). They will take serum samples as input. Subsequent analyses are done by university-employed scientists with special knowledge of multi-omics analyses.

### 5.5 Plans for collection and storage of biological specimens for molecular analyses

Blood samples will be drawn by medical laboratory technicians and prepared by laboratory technicians with special education in the required methodology. Tumor specimens will be collected by hospital-employed surgeons during the operation and immediately frozen in RNAlater at minus 20 degrees Celsius (https://www.thermofisher.com/no/en/home/brands/product-brand/rnalater.html) according to the manufacturer's instructions. PAX tubes for subsequent RNA extraction will be stored at minus 80 degrees Celsius. All samples will be stored in approved biobanks at Bærum Hospital in secure, environmentally controlled, and monitored (manual and automated) areas in accordance with the labeled storage conditions, with access limited to the investigator and authorized site staff. The samples will only be transferred to approved biobanks at Oslo University Hospital at the time of further processing and sequencing.

### 5.6 Dropout

When an enrolled participant later retracts the consent and leaves the study, does not show up to the scheduled appointments, is lost to follow-up or must be taken out of the study due to disease progression or death, it is considered a dropout. To reduce the dropout rate, all participants are thoroughly informed of the study requirements prior to inclusion. The study nurses are in close contact with the enrolled participants throughout the study period until the final endpoint measurement to ensure participant study completion. However, a small number of dropouts are inevitable. In case of a dropout, the time and reason for the dropout will be registered, and the participant will be removed from the final analyses. The number and reason for dropouts will be stated in the final study report.

#### 5.6.1 Participant discontinuation/withdrawal from the study

– A participant may withdraw from the study at any time without providing any reason. If a participant withdraws from the study, the participant may request destruction of any samples taken and not tested.
– A participant may be withdrawn at any time at the discretion of the investigator for safety reasons. This includes but is not limited to; death, disease progression, symptoms requiring urgent surgery such as bleeding or bowel obstruction, and cardiopulmonary adverse effects upon intervention such as syncopation or falls during exercise training.

#### 5.6.2 Lost to follow up

If an enrolled participant fails to show up at scheduled appointments for testing or exercise or refuses to participate in the intervention program or to provide the required blood samples, the study nurse will attempt to contact the participant and reschedule the missed visit and counsel the participant on the importance of maintaining the assigned visit schedule. If the participant repeatedly fails to return for scheduled visits or is unreachable for the study nurse, the participant is withdrawn from the study and is considered lost to follow-up. The contact attempts will be documented in the participant's medical record. In case of an unreachable participant, despite several contact attempts, the investigator will send a letter to the participant with information about the withdrawal.

## 6 Statistical methods

### 6.1 Primary endpoint

For the primary estimand with primary endpoint, change from baseline to timepoint 2 and 3, the following 1-sided hypothesis is planned to be tested for digital home-based prehabilitation vs. hospital-based prehabilitation. The mean treatment difference is defined as *μ* = [6 MWT result (meters) after digital home-based prehabilitation minus 6 MWT result (meters) after hospital-based prehabilitation].

The primary aim is to show that the digital prehabilitation program is not unacceptably worse than the hospital-based program, using a non-inferiority margin of 34 meters for the difference of the means.

Non-inferiority margin: d = 34 meters.

H0: μ ≥ d against Ha: μ < d.

The rationale for the non-inferiority margin is described in pt 4.4.1 and 4.5.

### 6.2 Cost-effectiveness analysis

To address missing data on resource use and EQ-5D-5L scores, Multivariate Imputation by Chained Equations (MICE) will be implemented (80). Multiple imputations will be combined with nonparametric bootstrapping to account for skewness, non-normality, and the correlation between costs and QALYs (81). Linear regression models will be fitted to each bootstrapped dataset to obtain adjusted estimates of mean total costs and QALYs for each treatment arm. These regression models will adjust the mean total costs and QALYs for baseline values and patient characteristics, such as age and gender (63). Total and incremental adjusted costs and QALYs will be summarized by presenting means, standard errors, and 95% confidence intervals. Uncertainty about the ICER will be presented by constructing cost-effectiveness acceptability curves and frontier, indicating the probability of the optimal intervention being cost-effective given a range of different willingness-to-pay thresholds (82). The Expected Value of Perfect Information (EVPI) will be computed to provide an upper bound on the potential value of conducting further research to reduce uncertainty (83).

## 7 Dissemination

The results from the dHOPE study will be published in international peer-reviewed scientific journals and presented at scientific meetings. Authorship will be determined by mutual agreement and in line with the International Committee of Medical Journal Editors’ authorship requirements.

The dHOPE study is a joint effort between patient groups, academia, clinics and commercial enterprises and is designed to allow for improved preoperative optimization of cancer patients and the successful deployment of novel medical device software (MDSW) aiding clinicians in personalization of prehabilitation programs, evaluation of operability of cancer patients, and preoperative decision-making. A clinical performance evaluation of the MDSW is anticipated for CE marking and the deployment of the software within 1 year following the completion of the proposed project and initial market entry within 1–3 years post-completion of the study. Diverse end-user scenarios, including hospitals, private clinics, and rehabilitation centers, are envisaged. To distribute the software and generate value beyond the scientific benefit of the study, a strategic business plan will be developed focusing on scalability and sustainability. Any successful MDSW will be available via subscriptions to health institutions.

## 8 Discussion

Clearly cancer patients scheduled for surgery have different starting vulnerabilities. They can present different degrees of fitness, frailty, and habits. Some have never been to a hospital before, while others have numerous comorbidities. It is reasonable to believe that the different patients, depending on their individual vulnerability, require tailor-made individual prehabilitation programs. Some may require a particular focus on cardiovascular exercise, while others need to develop their muscle strength. Some may need to emphasize nutrition and protein intake, while others need help with smoking cessation. Their individual needs must be addressed by an evaluation of their total situation, the type of tumor, the scheduled operation, and their desired outcome. For some patients, 5-year survival may be critical, while others value independence for a shorter period over long-time survival. To meet the patients' individual needs and desires we are obliged to personalize the prehabilitation programs as no standard program will be able to meet all the different needs. However, implementing personalized medicine in the public sector is still in its infancy. In the context of dHOPE, these challenges will be addressed by (i) integrating the information of many different biomarkers of vulnerability and surgery recovery together and including novel ones researched in the scope of this project, (ii) applying advanced explainable AI for intercorrelated analysis to integrate these data, (iii) producing an accurate and clinically validated measure of surgery recovery potential and (iv), enhanced patients' tailored recommendations to develop personalized prehabilitation programs to improve surgical outcome and maximize postoperative recovery.

A well-functioning prehabilitation program is a cost-effective and non-invasive approach that preserves functional capacity and quality of life, reduces complications, and dovetails with the wishes of cancer patients and the needs of society. A digital program opens for prehabilitation of all patients, despite living in rural areas far from hospitals. Furthermore, citizens should actively participate in their own health. Health literacy means that patients have good and fast access to personalized health information and the opportunity to participate proactively in their own treatment. The vision of dHOPE is to provide and integrate the personalized prehabilitation program within new digital decision software to (i) facilitate clinician-patient interaction and empower patients to take responsibility for their own health and (ii) create a more effective framework for healthcare professionals. Thus, dHOPE may identify subpopulations of patients who may benefit most from tailored interventions, leading to improved surgical outcomes and patient recovery. These subpopulations may be characterized by gender, age, frailty or comorbidities and will contribute to advancements in the field of patient-centric healthcare delivery. Intriguingly, results from dHOPE will be applicable to all kinds of surgery, not limited to cancer surgery, and is easily transferable to oncological treatment (e.g., prior to chemotherapy) and treatment of a variety of medical- and psychiatric diseases.
